# A 4‐year study of the proportional distribution of male reproductive organ abnormalities in cattle slaughtered at Nyagatare abattoir, Eastern Rwanda

**DOI:** 10.1002/vms3.69

**Published:** 2017-07-11

**Authors:** Erick Kandiwa, Leoncie Nyirakunzimana, Gervais Habarugira, Borden Mushonga, Alaster Samkange

**Affiliations:** ^1^ Department of Biomedical Sciences School of Veterinary Medicine Faculty of Agriculture and Natural Resources University of Namibia Windhoek Namibia; ^2^ School of Animal Sciences and Veterinary Medicine College of Agriculture Animal Sciences and Veterinary Sciences University of Rwanda Nyagatare Rwanda; ^3^ Department of Veterinary Clinical Studies School of Veterinary Medicine Faculty of Agriculture and Natural Resources University of Namibia Windhoek Namibia

**Keywords:** cryptorchidism, hypoplasia, scrotal hernia, orchitis, fertility, indigenous, exotic

## Abstract

Male reproductive performance has more impact on overall herd productivity than in the female. In order to assess herd productivity in cattle in Nyagatare, Eastern Rwanda, the proportional distribution of male reproductive organ abnormalities was investigated in 3087 bulls slaughtered over a 4‐year period. The aim of the study was to investigate the proportional distribution of male reproductive organ abnormalities in exotic and indigenous bulls slaughtered over a 4‐year period at Nyagatare abattoir in Eastern Rwanda. Positioning of the testicles was observed and recorded as the animals were assembled before slaughter. After slaughter, the internal and external reproductive organs of all bulls were removed, labelled and analysed for pathology. Significantly more indigenous (79.01%) than exotic (20.99%) animals were slaughtered (*P* < 0.05; *N* = 3 087). Overall occurrence of abnormalities was significantly higher in exotic (20.83%) than in indigenous (10.33%) animals (*P* < 0.05). Overall, abnormal location of testicles was the most common abnormality (4.08%) followed by abnormal prepuce and penis (2.33%), orchitis (1.94%), enlarged vesicular gland (0.91%), scrotal hernia (0.87%), unilateral cryptorchidism (0.81%), testicular hypoplasia (0.81%) and hydrocoele (0.78%). Abnormal location of testicles, abnormal prepuce and penis, orchitis, testicular hypoplasia and enlarged vesicular glands had significantly higher prevalence in exotic animals than in indigenous animals (*P* < 0.05). There was no significant difference in the prevalence of unilateral cryptorchidism, hydrocoele and scrotal hernia between the exotic and indigenous breeds. The encountered reproductive abnormalities result in poor herd fertility manifesting as low first‐service conception rates, prolonged breeding seasons and reduced weaning weights accompanied by inevitable financial losses in beef production. The observed high proportion of male reproductive abnormalities in exotic breeds might offset the professed benefits from introduction of these exotic breeds with the aim of improving productivity.

## Introduction

Nyagatare abattoir is located in Nyagatare district, the largest and second most‐populous district in Rwanda with about 243 people/km^2^. In 2012, a total of 340 000 cattle were slaughtered in Rwanda, and in 2014, the total cattle population of cattle in the country was only 1.14 million (Ministry of Finance and Economic Planning 2014). Most cattle in Nyagatare district are reared extensively in multiple‐sire herds on rangelands which make up only 7.4% of the total agricultural land (Ministry of Finance and Economic Planning 2014). By November 2013, Rwanda's abattoirs were able to supply only 7.59 kg meat per person per year thus falling far short of FAO recommendation of 30 kg meat per person per year (FAO, 2013). Even though most of the country receives reasonably high annual precipitation (950 mm) spread over about 133 days to ensure favourable pastures, there is still need for improvements in beef production in the country at large (World Meteorological Organization, 2015). This can be achieved through increased herd fertility, which in part can be achieved by culling bulls that are not breeding sound from the breeding stock. It has been argued that fertility of the bull has a greater impact on herd fertility (Tsakmakidis *et al*. 2010) as the bull serves about 30 animals per breeding season (Yimer *et al*. 2011). Since country‐wide breeding soundness evaluations are expensive and difficult to implement, this study aimed to assess the prevalence of male reproductive abnormalities so as to guide any form of proposed intervention measures aimed at dealing with their effect on herd fertility.

Male reproductive abnormalities can result in subfertility, infertility or sterility of the bull (Eshetu *et al*. 2016). Subfertility is a temporal infertility of a prolonged duration (Gnoth *et al*. 2005). In beef bulls, it is a worldwide phenomenon occurring in 20% of bulls, mainly due to poor semen quality and the inability of bulls to serve. Subfertility directly results in low conception at first‐service, which prolongs the breeding season (Mukhopadhyay *et al*. 2010). However, every 21 days of the breeding season that a cow is open results in a 22–28 kg loss in weaning weight of the calf she finally conceives in herds where blanket weaning is practised. Thus, a subfertile bull would result in financial losses due to reduced weaning weights a year later (Boligon *et al*. 2010; Inchaisri *et al*. 2010; Mukhopadhyay *et al*. 2010). Thus, in the beef industry, significant economic losses are due to delayed conception (Hopkins 2007). Due to the overlap of fertile and subfertile bulls in multiple‐sire pasture breeding, acceptable pregnancy rates are achieved when breeding pressure is low and the breeding season is prolonged. Another drawback in multiple‐sire herds is the increased risk of disease spread because 80% of the cows are serviced by two or more bulls during one oestrous period (Barth 2007). There is increasing economic pressure for highly efficient beef production through the implementation of short breeding seasons, use of high bull to female ratios, use of single‐sire breeding groups and thorough unbiased breeding soundness evaluations undertaken by veterinarians. (Hopkins 2007). In the long term, tolerance of low herd fertility leads to reduced productivity. To be fertile, a bull requires freedom from disease, good libido, physical soundness and good semen quality (Yimer *et al*. 2011). The number of matings a bull is willing and able to perform is hindered by injuries, penile deviations and the presence of an older dominant bull. The status of the male reproductive organs thus directly and indirectly affects breeding soundness in bulls (Barth & Ominski 2000). Gross lesions of the male reproductive tract that results in subfertility, infertility or sterility include scrotal wound, scrotal torsion, urethral obstruction, orchitis, testicular haematoma, testicular hypoplasia, epididymitis and cryptorchidism (Eshetu *et al*. 2016). Other gross abnormalities of the male reproductive tract include preputial lacerations, penile haematomas, preputial prolapse, preputial‐penile adhesions and other anatomical/structural abnormalities of the prepuce and penis and accompanying inflammation (Anderson 2008).

Abnormally located testicles refer to a condition in which the scrotum is placed too far forward or too far behind its normal location in the inguinal region. This condition often result in impaired thermoregulation (and thus reduced semen quality) and increased incidence of traumatic damage. This condition must be distinguished from cryptorchidism. Some breeding herds reportedly had low incidence (0.3%) of abnormally located testicles possibly due to the exclusion of most affected bulls at breeding soundness evaluation (Chenoweth 2015).

Cryptorchidism is the failure of descent of one or both testes into the scrotum as a result of developmental anomalies, mechanical obstruction or hormone deficiency. As a result, the testes become lodged at the external inguinal ring, in the inguinal canal, at the internal inguinal ring or in the abdomen. Cryptorchidism can either be unilateral or bilateral with the former being 75% more common than the later. Cryptorchidism reportedly occurred in 0.17% in some herds (Jean *et al*. 1992) and in 1.4% in others (Godfrey & Dodson 2005). Investigators generally agree that this condition is heritable (Amann & Veeramachaneni 2006, 2007; Smith & Sherman 2009; Adeyeye & Wakkala 2013; Mahmud *et al*. 2015), and thus, it is best for the cryptorchid bulls to be removed from the breeding stock. Although slaughtering of cryptorchid bulls has been practised over a long time, it is becoming clear that this strategy alone is not achieving the intended results. It has been reported that exposure of an embryo to oestrogenic substances *in utero* may also play a role in the pathogenesis of this condition (Newbold 1995; Cederroth *et al*. 2007).

Preputial lacerations, penile haematomas, preputial prolapse, preputial‐penile adhesions and other anatomical/structural abnormalities of the prepuce and penis and accompanying inflammation result in reluctance of bulls to breed and inability of bulls to effectively copulate with cows and heifers (Anderson 2008). Some highly active breeding groups of bulls have reported 100% incidence of penile and preputial abnormalities that resulted from increased the breeding pressure (Karle *et al*. 2010).

In male cattle, *Brucella abortus* (Megid *et al*. 2010) and *Histophilus somnus* (Díaz‐Aparicio *et al*. 2009) are typically is associated with seminal vesiculitis, orchitis and epididymitis. Unilateral orchitis and epididymitis usually result in degenerative changes in the normal testis/epididymis through the elevated temperatures of inflammation and pressure necrosis resulting from accompanying oedema (Gnemmi & Lefebvre 2009; Bousmaha & Khoudja 2012). The presence of such inflammatory conditions in male cattle (though not only due to brucellosis) reduces the mating ability of the bulls. The zoonotic significance of brucellosis ought to be considered as some African customs support consumption of inadequately cooked male gonads during ritual slaughters (Miller 1985). Studies by other workers have revealed that 17.7% of bulls had seminal vesiculitis (Gomes *et al*. 2000).

Testicular hypoplasia is agreeably a heritable phenomenon whose prevalence generally varies between 2.5–3.3% in some herds and 0.5% in others (Godfrey & Dodson 2005). These variations were possibly due to different breeding practices. Recently, a study of testicular hypoplasia in 68 bulls in Mato Grosso, Brazil and reported an incidence of 11.8% (Oliveira *et al*. 2011). This condition results from a hereditary reduction in number and size of functional germ cells in seminiferous tubules of germ cells rather than a total lack of them (Forster 2012).

Hydrocoele are pathological accumulations of serous fluid in the tunica vaginalis which can be congenital or acquired (a result of altered rate of secretion of fluid by the vaginal tunic, altered resorption of serous secretions by veins and lymphatics of the spermatic cord, testicular trauma, inflammation, torsion, hernia or ascites) but invariably leading to testicular swelling, redness, pain and eventually leading to testicular degeneration as result of pressure necrosis and impaired thermoregulation (Yimer *et al*. 2011). Most (85%) hydrocoele have been observed to resolve after 120 days (Shore *et al*. 1995). There is no literature on the incidence of hydrocoele in bulls possibly due to the fact that successful definitive diagnosis is achievable by ultrasonography (Gnemmi & Lefebvre 2009; Abu‐Seida 2012).

Inguinal hernias occur when gastrointestinal viscera descend past the internal inguinal ring through the inguinal canal into the vaginal sac. These can become scrotal hernias when ‘migrating’ viscera extend past the external inguinal ring into the scrotum (Ayars 2006). The underlying cause of inguinal/scrotal hernias is usually congenitally enlarged vaginal rings (Chenoweth 2015). These hernias can be hereditary, congenital or acquired, painful or non‐painful and may rupture. They always lead to the condemnation of bulls from the breeding stock (Gnemmi & Lefebvre 2009). There is, however, no literature on prevalence of scrotal/inguinal hernias in bulls. Other less common afflictions of the male reproductive system include vesicular adenitis and vasculitis (Martínez *et al*. 2008). Therefore, the purpose of this study was to investigate the proportional distribution of male reproductive organ abnormalities in exotic and indigenous bulls slaughtered over a 4‐year period at Nyagatare abattoir in Eastern Rwanda.

## Materials and methods

All the non‐castrated male cattle (aged between 1 and 6 years old) slaughtered at Nyagatare Abattoir from August 2011 to August 2015 were the basis of this study. A total of 3087 intact male bovines (2439 indigenous and 648 exotic) were slaughtered during the duration of the study, representing 79.01% and 29.99% proportions for indigenous (Ankole) and exotic (Friesian) animals respectively (Table 1).

**Table 1:** Overall distribution of animals slaughtered at abattoir

Breed of animals	Number of animals slaughtered	Proportion (%)
Indigenous	2439	79.01
Exotic	648	20.99
Total	3087	100.00

For this study, any encountered indigenous/exotic cross breed males were identified and recorded within the exotic group. In addition, intact males in this study were not necessarily bulls that had been selected for breeding. Nyagatare abattoir is the only destination for cattle due for slaughter in Nyagatare district, Eastern Rwanda. The bulk of these cattle were reared on open range in multiple‐sire herds by the communal farmers of Nyagatare district. Positioning of the testicles was observed and recorded as the animals were assembled before slaughter by the veterinarian and inspectors on duty. After slaughter, the internal and external reproductive organs of all intact males were removed and properly labelled to await further analysis. Pathological analysis of the extracted reproductive organs was performed by the researchers with the aid of the veterinarian on duty. Abnormalities were recorded and analysed using the Statistical Package for Social Sciences (SPSS) version 16.

All the relevant guidelines as prescribed by the Rwandan authorities were followed in the collection of the samples. There were no ethical issues involved in this study because the samples were from the abattoir.

## Results

The results of the study are summarized in the tables below (Tables 2, 3 and 4). Of the 3087 animals which were slaughtered, a total of 387 had reproductive abnormalities, of which 252 (10.33%) were indigenous and 135 (20.83%) were exotic (Table 2).

**Table 2:** Overall distribution of bovine male reproductive abnormalities between breeds

Breed of animals	Animals with normal organs	Animals with abnormal organs	Total number of animals slaughtered	Proportion of organs with abnormalities (%)
Indigenous	2187	252	2439	10.33
Exotic	513	135	648	20.83
Total	2700	387	3087	12.54

**Table 3:** Distribution of bovine male reproductive abnormalities in the total population of animals slaughtered

Type of abnormality	Number of animals with abnormality	Overall prevalence of abnormality (%)
Abnormal location of testicles	126	4.08
Abnormal penis/prepuce	72	2.33
Orchitis	60	1.94
Unilateral cryptorchidism	25	0.81
Testicular hypoplasia	25	0.81
Hydrocoele	24	0.78
Enlarged vesicular glands	28	0.91
Scrotal hernia	27	0.87
Total animals slaughtered	387	12.53

**Table 4:** Summary statistics on prevalence of bovine male reproductive abnormalities between indigenous and exotic animals

Type of abnormality	Number of indigenous cases	Number of exotic cases	Chi square value	Total number of animals in test (*N*)	*P*‐value
Abnormal location of testicles	81	45	21.22	2826	0.00^a^
Abnormal penis and prepuce	45	27	15.30	2772	0.00^a^
Orchitis	42	18	4.57	2760	0.03^a^
Unilateral cryptorchidism	17	8	2.71	2725	0.10^b^
Testicular hypoplasia	15	10	7.04	2725	0.01^a^
Hydrocoele	16	8	3.16	2724	0.08^b^
Enlarged vesicular glands	18	10	4.99	2728	0.03^a^
Scrotal hernia	18	9	3.55	2727	0.06^b^
Overall abnormalities	252	135	51.49	3087	0.00^a^

a Significant difference in prevalence of abnormality between exotic and indigenous male bovines (*P* < 0.05).

b No significant difference in prevalence of abnormality between exotic and indigenous male bovines (*P* > 0.05).

The eight different abnormalities which were detected as well as their proportions are shown in Table 3. These ranged from hydrocoeles, which had the lowest prevalence of 0.78%, to abnormal location of testicles which had a prevalence of 4.08%.

The summary statistics were also calculated and are shown in Table 4. Overall, there were significant differences in the prevalence of five of these abnormalities between the two categories of animals.

The occurrence of abnormalities was significantly higher (*P* < 0.05) in exotic (20.83%) than in indigenous (10.33%) animals. The abnormal location of testicles was the most common abnormality (4.08%) followed by abnormal prepuce and penis (2.33%), orchitis (1.94%), enlarged vesicular gland (0.91%), scrotal hernia (0.87%), unilateral cryptorchidism (0.81%), testicular hypoplasia (0.81%) and hydrocoele (0.78%). However, abnormal location of testicles, abnormal prepuce and penis, orchitis, testicular hypoplasia and enlarged vesicular glands had significantly higher prevalence in exotic animals than in indigenous animals (*P* < 0.05). There was no significant difference in the prevalence of unilateral cryptorchidism, hydrocoele and scrotal hernia between the exotic and indigenous breeds.

The results in Table 5 above show that, overall, the exotic animals were 2.7 times more likely to be affected by gonadal abnormalities than the indigenous animals. These gonadal abnormalities ranged from orchitis which was 1.9 times more likely to occur in exotic animals than in indigenous males to testicular hypoplasia which was 2.9 times more likely to occur in exotic animals than in indigenous animals.

**Table 5:** Summary of odds ratios of bovine male reproductive abnormalities between indigenous and exotic animals

Abnormality	Exotic animals positive	Exotic animals negative	Indigenous animals positive	Indigenous animals negative	Odds Ratio
Abnormal location of testicles	45	468	81	2106	2.5
Abnormal penis and prepuce	27	486	45	2142	2.6
Orchitis	18	495	42	2145	1.9
Unilateral cryptorchidism	8	505	17	2170	2
Testicular hypoplasia	10	503	15	2172	2.9
Hydrocoele	8	505	16	2171	2.1
Enlarged vesicular glands	10	503	18	2169	2.4
Scrotal hernia	9	504	18	2169	2.2
Overall abnormalities	135	378	252	1935	2.7

## Discussion

The results from this study show that more indigenous than exotic male animals were brought to Nyagatare Abattoir for slaughter over the 4‐year duration. This distribution, however, cannot be used to infer on the distribution of indigenous and exotic animals in cattle population of Nyagatare district. The study provides vital information on the prevalence of male reproductive organ abnormalities over the 4‐year period.

The prevalence of abnormally located testicles was the most common condition in both indigenous and exotic animals and the study revealed that this occurred in relatively similar proportions regardless of breed. A plausible inference for such findings is that the abnormal location of testicles in the animals under study was not breed related but was due to other factors (environmental or husbandry) common to both indigenous and exotic animals. Abnormally located testicles predispose bulls’ testicles to trauma resulting in high temperatures (leading to testicular degeneration and reduced sperm motility) and testicular inflammation and pain (Rhynes & Ewing 1973; Rasooli *et al*. 2010). Since this condition is highly heritable, culling of bulls with this abnormality is highly recommended to achieve long term improvements in herd fertility (Amann & Veeramachaneni 2007). The high prevalence of this condition in cattle in Nyagatare must not be taken lightly, considering that the Government is focusing on crossing of indigenous and exotic breeds as a way of increasing productivity of both meat and milk, given that land is a very limited resource in Rwanda.

The prevalence of cryptorchidism at 0.81% is also worrisome as the condition is considered heritable (Amann & Veeramachaneni 2006, 2007; Smith & Sherman 2009; Adeyeye & Wakkala 2013; Mahmud *et al*. 2015). Other workers have previously argued that exposure of a male embryo/foetus to an oestrogen rich environment *in utero* during the development of the reproductive tract may also increase the incidence of cryptorchidism (Newbold 1995; Cederroth *et al*. 2007).

Prevalence of orchitis and abnormal prepuce/penis, although less common than abnormally located testicles, was more common than the rest of the other abnormalities encountered. Orchitis invariably leads to increased scrotal temperatures that culminate in testicular degeneration (and usually testicular hypoplasia) of any remaining testicular tissue and thus directly affecting fertility (Schuppe *et al*. 2008).

In the light of the aforementioned, it is evident that the prevalence of male reproductive abnormalities in intact male cattle destined for slaughter at Nyagatare district is a cause for concern for Rwanda, if the results of this study provide the correct key‐hole view of the prevalence of the problem in the whole district and/or country. If this study is extended to other districts, an overall impression of the problem would be gained and appropriate interventions instituted.

## Source of funding

This study was funded by University of Rwanda.

## Conflict of interest

The authors declare that they have no conflict of interest.

## Ethics statement

No ethical approval was required because the study made use of abattoir specimens.

## Contribution

EK did the designing of the study, the statistical analysis and the final manuscript write up and editing. GH contributed to the designing of the study, supervision of the project, and writing of the final version of the manuscript. LN did the data collection and writing of the draft manuscript. BM did the data analysis, the write up of the draft and editing of the final version of the manuscript. AS did the designing of the studies, write‐up of the draft, editing the final version and coordinated the publication of this manuscript. All authors read and approved the final manuscript.
